# Supplementary material for: Learning curve and surgical outcome of robotic assisted colorectal surgery with ERAS program
Source: Sci Rep. 2022 Nov 29;12:20566. doi: 10.1038/s41598-022-24665-w (PMC9709162; doi:10.1038/s41598-022-24665-w)
Supplement: Supplementary file 1 — Supplementary Information 1. [file 41598_2022_24665_MOESM1_ESM.zip › LSC Raw data-Ñ[▒K/063/N_ñw╝╨░O▒Kñσ.pdf]

W52 / W52

臺中榮民總醫院
手術室護理記錄

[redacted]

基本資料

- 室別： 二醫手術室-31室，手術科別： CRS，手術類別： 常規，日期： 2020/01/16
- 進入時間： 1245 ； 麻醉開始時間： 1250 ； Time Out時間： 1330 ； 劃刀時間： 1330 ； 手術結束時間： 1550 送出時間： 1610 ；
- 麻醉方式： GE
  診斷：RECTAL CA WITH LIVER METS
  手術名稱：Laparoscopic Anterior resection and anastomosis - malignant
  術前預防性抗生素：
  - 抗生素名稱 ： Veterin 1GM IVD/由麻醉護理執行
  - 時間： 1330
  - 上次簽章: 彭馨慧

  ，追加預防性抗生素：
- 手術醫師： 蔣鋒帆
- 麻醉醫師： VS 賴慧卿
-
  - 刷手護士A ： 林昱君，時間： 1245
  - 流動護士A ： 彭馨慧，時間： 1245，接替流動一： 顏育?，時間： 1500

皮膚狀況

A-V Shunt： 無
術前： 完整
記錄者： 上次簽章: 彭馨慧
術後： 完整
記錄者： 上次簽章: 顏育？

手術準備

手術臥式 單一手術臥式

-
  - 手術臥式： 截石臥式
  - 使用附件： 溫毯 38 ℃，頭圈，腳架
  - 皮膚準備： 10%Aq Betadine，Hibiscrub，其它： 2%Chlohexcidine
  - 電燒： 使用記錄者： 上次簽章: 彭馨慧

止血帶T： 無

植入物

- 植入物名稱/號碼/數量/部位：Ethicon ligaclip REF ER420/colon/20釘
  Ethicon Endogiaclip/?釘/colon
  Surgiwrap/100*130*0.02mm/壹/pelvic

，記錄者： 上次簽章: 顏育？

標本送檢

- 細菌培養 無
- 細胞學 無
- 冰凍切片 無
- 病理標本
  組織名稱一：rectum
  ，
  組織名稱二：IMA root
  ，
  組織名稱三：distalcurend
- 截肢 無

標本交付 標本： 參 袋，標本： 零 盒，標本： 零 瓶，細胞學： 零 管，Culture Tube： 零 管，被交付醫師： R 邱

俊凱，

- 記錄者： 上次簽章: 顏育？
- 時間： 1600

傷口術後敷料

傷口敷料： 傷口敷料： 無

- 請於圖上標示部位：

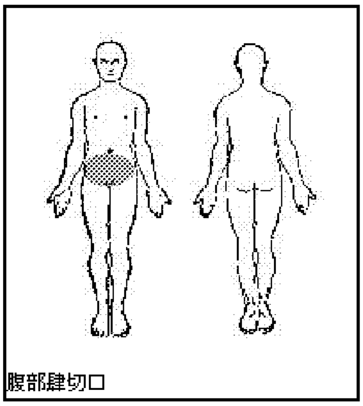

- 傷口敷料：menpor覆蓋

導管

導管	天數	導管資訊	插入日期	插入時間	更換日期	拔除日期	拔除時間	拔除原因	預計拔除/更換	預計拔除/更換日期
Foley(1)	1	號碼:16 Fr.; 部位:尿道;	2020/01/16	1300						
Sump NG Tube(1)	1	號碼:16 Fr.; 部位:胃;	2020/01/16	1250						
Endotracheal Tube(1)	1	號碼:6.5 mm.; 部位:氣管; 註記:fix22cm;	2020/01/16	1300						
Penrose Drain(1)	1	號碼:12 mm.; 部位:骨盆腔; 註記:左腹;	2020/01/16	1545						
A-line(1)	1	號碼:22 號; 部位:左手;	2020/01/16	1255						

用物計數

- 手術中出血量： 少 c.c.
- 手術中尿量： 400 c.c.
-
    - 其它： 無

用物計數： 多切口同時縫合

- 1.器械： 正確，2.縫針： 正確，3.刀片： 正確，4.紗布： 正確，5.腹部墊： 未使用，6.紗球： 未使用，7.PEANUTS ： 未使用，8.紗條： 未使用，9.絲帶： 未使用，10.針頭： 未使用，11.Vessel loop： 未使用，12.Boots： 未使用，13.Jelco： 未使用，14.棉條： 未使用，
    - 第一次：
        - 刷手護士 ： 林昱君
        - 流動護士： 上次簽章: 顏育？
        - 時間： 1535

    ,
    - 第二次：
        - 刷手護士 ： 林昱君
        - 流動護士： 上次簽章: 顏育？
        - 時間： 1545

    ,
    - 第三次：
        - 刷手護士 ： 林昱君
        - 流動護士： 上次簽章: 顏育？
        - 時間： 1555

備註

備註： NIL

- 記錄者： 上次簽章: 顏育？
- 時間： 1610

填寫護理人員 顏育？

正式摘要

W52 / W52

doctorRank:B
